# Design of OMC-Sagnac Loop Using PDMS and Different Package Structures to Improve Sensing Performance and Optimize the Ill-Conditioned Matrix

**DOI:** 10.3390/s23104655

**Published:** 2023-05-11

**Authors:** Shumao Zhang, Yang Yu, Xiaoyang Hu, Qiang Bian, Dongying Wang, Junjie Weng, Jianqiao Liang, Linyi Wei, Peng Jiang, Hong Luo, Linfeng Yang, Junbo Yang, Zhenrong Zhang

**Affiliations:** 1 Key Laboratory of Disaster Prevention and Structural Safety of Ministry of Education, School of Computer, Electronics and Information, Guangxi University, Nanning 530004, China; 2013391089@st.gxu.edu.cn (S.Z.);; 2 College of Advanced Interdisciplinary Studies, National University of Defense Technology, Changsha 410073, China; 3 College of Sciences, National University of Defense Technology, Changsha 410073, China; 4 College of Meteorology and Oceanography, National University of Defense Technology, Changsha 410073, China; 5 Institute for Measurement Systems and Sensor Technology, Technical University of Munich, 80333 Munich, Germany; 6 Changsha Sensintel Information Technology Co., Ltd., Changsha 410201, China; 7 Guangxi Key Laboratory of Multimedia Communications and Network Technology, Guangxi University, Nanning 530004, China; 8 Guangxi Key Laboratory of Disaster Prevention and Engineering Safety, Guangxi University, Nanning 530004, China

**Keywords:** OMCSL, ill-conditioned matrix, sensor structure optimization, machine learning method

## Abstract

In the process of ocean exploration, highly accurate and sensitive measurements of seawater temperature and pressure significantly impact the study of seawater’s physical, chemical, and biological processes. In this paper, three different package structures, V-shape, square-shape, and semicircle-shape, are designed and fabricated, and an optical microfiber coupler combined Sagnac loop (OMCSL) is encapsulated in these structures with polydimethylsiloxane (PDMS). Then, the temperature and pressure response characteristics of the OMCSL, under different package structures, are analyzed by simulation and experiment. The experimental results show that structural change hardly affects temperature sensitivity, and square-shape has the highest pressure sensitivity. In addition, with an input error of 1% F.S., temperature and pressure errors were calculated, which shows that a semicircle-shape structure can increase the angle between lines in the sensitivity matrix method (SMM), and reduce the effect of the input error, thus optimizing the ill-conditioned matrix. Finally, this paper shows that using the machine learning method (MLM) effectively improves demodulation accuracy. In conclusion, this paper proposes to optimize the ill-conditioned matrix problem in SMM demodulation by improving sensitivity with structural optimization, which essentially explains the cause of the large errors for multiparameter cross-sensitivity. In addition, this paper proposes to use the MLM to solve the problem of large errors in the SMM, which provides a new method to solve the problem of the ill-conditioned matrix in SMM demodulation. These have practical implications for engineering an all-optical sensor that can be used for detection in the ocean environment.

## 1. Introduction

With the development of ocean exploration, there is a need for more sensitive and timely information extraction from seawater. The temperature and pressure of seawater become crucial hydrological parameters that can indirectly infer key information, such as resource distribution, ocean current trends, etc. Therefore, the multiparameter and high-precision rapid measurements of sensors have an important impact on the study of physical, chemical, and biological processes in seawater.

Traditionally, temperature, salinity, and depth monitoring of the ocean is achieved by electrical conductivity–temperature–depth (CTD) sensors [1,2]. However, electrical sensors often face problems of high price, electromagnetic interference, and high requirements for water tightness. In contrast, optical sensors not only solve the problems above, but also have outstanding advantages, such as low cost, ease of integration, immunity to electromagnetic interference, and no requirements for water tightness. Therefore, optical fiber sensors for marine detection are receiving more attention in recent decades. A large number of optic fiber temperature and pressure sensors have been proposed, such as the Mach–Zehnder interferometer (MZI) [3,4], fiber Bragg grating (FBG) [5], tapered fiber [6], Fabry–Pérot interferometer (FPI) [7], etc. In the process of measuring the temperature and pressure of seawater, researchers use various materials and special packaging methods to protect and sensitize sensors, and usually pick the sensitivity matrix method (SMM) to demodulate the matrix of multiphysical parameters. For example, Zhang et al. proposed and experimentally demonstrated a seawater temperature and pressure sensor realized by a polyimide (PI) tube-based Fabry–Pérot interferometer (FPI) together with a fiber Bragg grating (FBG). Benefiting from the higher thermo-optical coefficient and larger elasticity of the polymer than fused silica fiber, the sensitivity of the sensor is largely improved [8]. Lu et al. used an optical microfiber coupler encapsulated PDMS and received an order of magnitude higher sensitivity than the unencapsulated bare optical microfiber coupler, and used the sensitivity matrix method to demodulate temperature and pressure dual parameters [9]. All of the articles mentioned above utilize the sensitivity matrix method for demodulation; however, none of them take into account the issue of errors in the sensitivity matrix method, or the impact of temperature and pressure sensitivity magnitudes on these errors.

Therefore, when using the SMM to demodulate seawater temperature and pressure, sensitivity characteristics are basically the same between different interference peaks in a single sensor, leading to the ill-conditioned matrix problem of sensors. Under this problem, a small input error will produce a large output error, resulting in the systematic error being amplified to an unacceptable value when using the SMM. To solve this problem, researchers cascaded different sensors to alleviate the ill-conditioned matrix problem via the difference in sensing characteristics between different sensors. For example, Fang et al. achieved dual parametric demodulation of temperature and salinity by cascading TFBG and FBG, which obtained a temperature sensitivity of 11.60 pm/°C and a salinity sensitivity of 0.833 nm/RIU [10]. Jiang et al. achieved a response sensitivity of temperature and SRI of 11.83 pm/°C and 510.48 nm/RIU, respectively, by cascading two TFBGs with different characteristics [11]. Although the above sensors alleviated the ill-conditioned matrix problem to some extent, they used some physical methods to deal with the sensors, and have shortcomings such as poor integration and low sensing sensitivity, which are challenging needs to meet for the monitoring of the natural marine environment.

The optical microfiber coupler (OMC), as a basic passive optical device, has the advantages of large-scale evanescent field transmission characteristics, low loss, and tiny size, which provide an excellent optical platform for development, sensing, testing, and application of optical functional devices. In addition, the sensing characteristics of the OMC can be improved by certain package designs. Different package structures are expected to change the magnitude of pressure sensitivity, so that the sensitivity difference between different interference peaks and the angle between the lines in the sensitivity matrix will increase, and finally, the ill-conditioned matrix problem will be effectively optimized. As a result, an OMC packaged in a specific package design is expected to achieve high-sensitivity dual parameter sensing and reduce demodulation errors.

Based on the above problems, this paper designs and fabricates three different packaging structures, V-shape, square-shape, and semicircle-shape, to optimize the ill-conditioned matrix problem, and uses PDMS to package the OMC into these structures. Then, the sensing characteristics of the OMC in different packaging structures are discussed. This paper also simulates the pressure response characteristics of the OMC in different package structures, and demonstrates the pressure and temperature response characteristics of the OMC in different package structures in experiments. It can be found that the pressure sensitivity of the OMC in V-shape, square-shape, and semicircle-shape package structures are 0.203 nm/MPa, 1.135 nm/MPa, and 0.674 nm/MPa, respectively, and temperature sensitivity are −0.766 nm/°C, −0.648 nm/°C, and −0.649 nm/°C, respectively. This phenomenon indicates that pressure sensitivity is more affected by the package structure, and temperature sensitivity is basically not affected by the package structure. This paper optimizes the ill-conditioned matrix problem of the sensor with the semicircle-shape package; this special package structure can significantly increase the angle between two intersecting lines in the sensitivity matrix. In addition, with an input error of 1% F.S., the temperature and pressure errors are calculated to be 0.69 °C and 0.36 MPa for the V-shape, 1.77 °C and 0.48 MPa for the square-shape, and 0.60 °C and 0.31 MPa for the semicircle-shape, respectively. This means that in the case of unavoidable input errors in practical applications, the semicircle-shape package structure can significantly reduce the errors caused by cross-sensitivity in the SMM, thus effectively optimizing the ill-conditioned matrix problem. Finally, this paper proposes using the MLM to solve the problem of large errors in the SMM, and also compares the demodulation errors of the SMM and MLM, in which the MLM has higher demodulation accuracy; however, the SMM has a simple structure and faster result output, so different methods should be selected according to actual engineering needs. In conclusion, this paper proposes to optimize the ill-conditioned matrix problem in SMM demodulation by improving sensitivity with structural optimization, which essentially explains the cause of large errors for multiparameter cross-sensitivity. In addition, this paper proposes to use the MLM to solve the problem of large errors in the SMM, which provides a new method to solve the problem of ill-conditioned matrix in SMM demodulation. These have practical implications for engineering an all-optical sensor which can be used for detection in the ocean environment.

## 2. The Fabrication and Sensing Principle

### 2.1. Design and Fabrication

**Figure 1:**
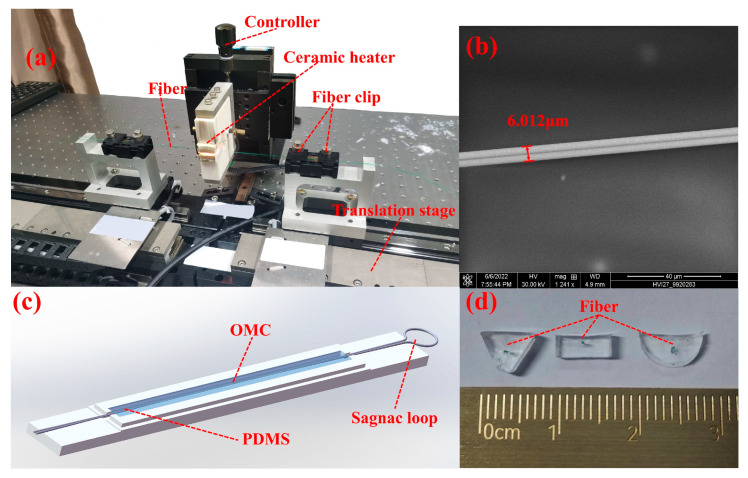
(**a**) Fiber taper system diagram; (**b**) scanning electron microscope (SEM) image of the OMC waist region; (**c**) schematic diagram of the OMCSL and PDMS structure; (**d**) cross-sectional view of the different package structures.

The proposed sensor OMCSL is made by drawing a standard single-mode fiber (corning, SMF-28e) into two equal radius sections with a fiber taper system (Figure 1a). The samples drawn by this taper system have the advantages of controlled structural parameters, strong mechanical properties, low insertion loss, etc. [12,13]. By observing the diameter of the waist region through a microscope before encapsulating, it can be found that the waist region of the OMCSL is similar after setting the structural parameters in the system with different drawing times. In this paper, a uniform waist diameter of the OMCSL is set to 6 µm (Figure 1b), and the coupling length is set to 7 mm.

Due to the high thermo-optical coefficient and good flexibility of PDMS [14], the OMCSL encapsulated by PDMS can be used for high-sensitivity temperature and pressure sensing. Encapsulating with PDMS can also improve structural stability and reduce the bending loss of the sensor. In this paper, the main agent (Sylgard 184, Dow Corning, Midland, Michigan, USA) is mixed with the curing agent at a ratio of 10:1, and then cured to obtain the encapsulated sensor [15]. Figure 1c shows the cross-section of PDMS after curing in different package structures.

### 2.2. Theoretical Analysis

**Figure 2:**
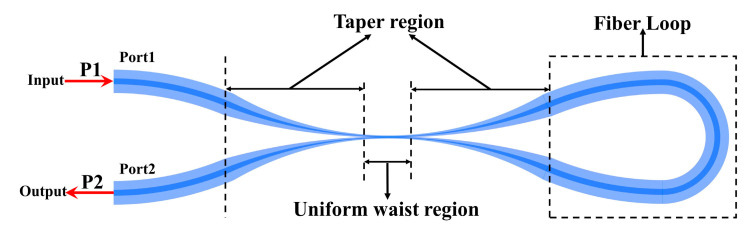
Schematic diagram of the OMCSL composition.

The structure of the OMCSL consists of a uniform waist region, two tapered transition regions, and a Sagnac loop, as shown in Figure 2. The part of complete coupling in the uniform waist region is called the strong coupling region, while the incomplete coupling part in the tapered transition region is called the weak coupling region, and different coupling coefficients correspond to the degree of coupling [16]. The Sagnac loop is responsible for reflecting the incident light back to the detection port. In addition, the uniform waist region and the tapered transition region are fully encapsulated in PDMS, as the primary sensing components in the mentioned structure.

In the weak coupling region, the coupling degree increases with the decrease of the fiber radius. At this time, the light field gradually penetrates the fiber cladding and the external medium, and the fiber cladding and the external medium can be regarded as the waveguide core and cladding, respectively. The coupling coefficient in the weak coupling region can be expressed as follows [17,18]:

(1) $$C_{WC}=\frac{2^{1/2}{\left(n_{2}^{2}-n_{3}^{2}\right)}^{1/2}}{n_{2}r\sqrt{\pi }V^{5/2}}\times {\left(V_{C}\right)}^{2},$$

where, $V=\frac{2\pi r}{\lambda }\sqrt{n_{2}^{2}-n_{3}^{2}}$ is the normalized frequency, $V_{C}=$ 2.405 is the cutoff normalized frequency, $n_{2}$ and $n_{3}$ are the refractive indices of the fiber cladding and external environment, respectively, and λ is the wavelength of the incident refractive index.

In the strong coupling region, the two fibers are completely fused. In this waveguide structure, the whole waveguide structure can be regarded as a kind of hybrid waveguide with two super modes and simplified to the case of the theory of local coupling [19]. The coupling coefficient in the strong coupling region can be expressed as follows [20,21]:

(2) $$C_{SC}=\frac{3\pi \lambda }{32n_{2}r^{2}}\cdot \frac{1}{{\left(1+1/V\right)}^{2}},$$

The coupling region only consists of the uniform waist area and two tapered transition regions. The two tapered transition regions have a small taper angle, and the coupling coefficient changes uniformly and slowly, so the coupling characteristics of the OMCSL can be analyzed by the local coupling mode theory. When the light with initial power $P_{1}$ is injected from port 1, the output power of port 2 can be described as [22]:

(3) $$P_{2}=P_{1}[1-\frac{1}{2}\sin^{2}(2\int_{0}^{L}C(\lambda ,n_{2},z)dz)],$$

where, $C\left(\lambda ,n_{2},z\right)$ represents the coupling coefficient at position z, and L represents the coupling length.

## 3. Simulation Results

### 3.1. Theoretical Analysis

This paper presents a numerical temperature sensitivity simulation of the OMCSL encapsulated by PDMS [23]. The principle of temperature sensing is based on the fact that the characteristic wavelength of the OMCSL spectrum changes with temperature, and PDMS, as the refractive index of the ambient material, will change at the same time. In this case, the PDMS solution with a concentration of 10:1 has a relationship between the refractive index and temperature, as follows [24]:

(4) $$n_{PDMS}(T)=-4.66\times 10^{-4}\cdot T+1.4176,$$

**Figure 3:**
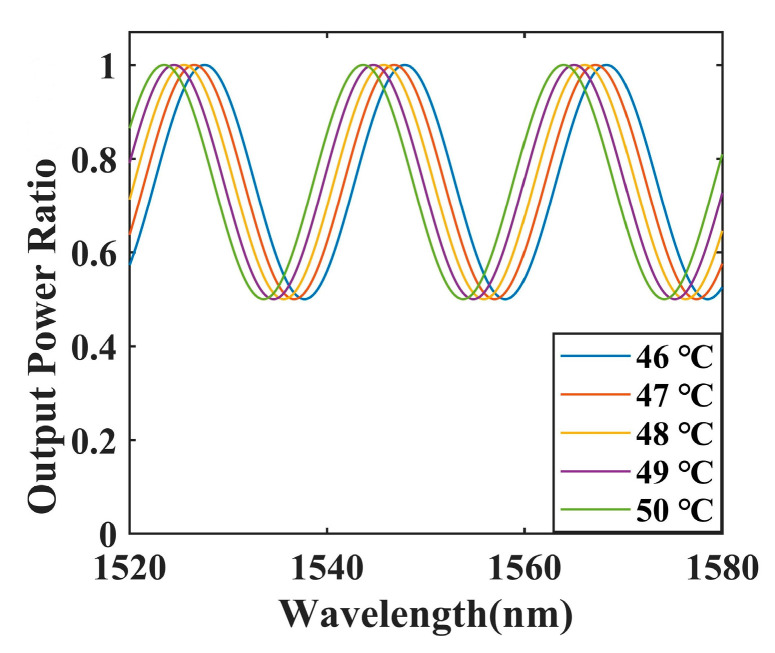
The resulting spectra of the encapsulated OMCSL with different temperatures.

According to the study related to temperature simulations, the input light intensity of port 1 is set to 1. Under these conditions, this paper conducted MATLAB numerical simulations of the sensor by combining the coupling coefficient formula with the PDMS thermo-optical coefficient formula. The temperature variation spectrum of the OMCSL is shown in Figure 3. As can be seen, the spectrum of the OMCSL blueshifts linearly as the temperature increases. This phenomenon can be attributed to the RI of the PDMS decreasing with the increasing temperature due to the negative thermo-optic coefficient of PDMS, so the spectral dips blueshift with the increasing temperature.

### 3.2. Analysis of Pressure Sensing Characteristics in Different Package Structures

This paper also designs three different package structures, V-shape, square-shape, and semicircle-shape, and simulates them to verify which package structure could maximize the force on the fiber (corresponding to the maximum fiber pressure sensitivity) in the OMCSL. The simulation structure designed in this paper is that PDMS in different structures will completely wrap the uniform waist region and the conical transition region of the OMCSL, and is cured into different shapes by the action of the slot. The same structural parameters are used for all three structures. The Young’s modulus and Poisson’s ratio of the PDMS were set to 7.5 × 10^5^ N/m^2^ and 0.495 [25], respectively, and those of the fiber were set to 7 × 10^10^ N/m^2^ and 0.17, respectively.

**Figure 4:**
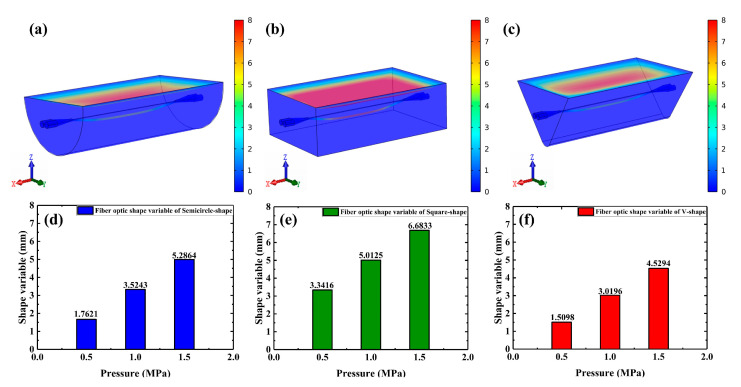
Force analysis diagram of (**a**) semicircle-shape, (**b**) square-shape, and (**c**) V-shape sensors at 1.5 MPa. Optical fiber deformation variables of (**d**) semicircle-shape, (**e**) square-shape, and (**f**) V-shape structures at different pressures.

All the slots of the three package structures used in the experiments are aluminum slots, which are considered to be rigid in the simulation, so that only the upper surfaces of the models are under pressure, and the other surfaces are restrained. In this design, the fibers pass through the center of the PDMS, and are equally long to the bottom surface in all three structures. As shown in Figure 4a–c, when pressure is applied to the uniform waist region of the fiber along the z-axis, different degrees of deformation are found in the fiber in the various structures.

The optical fiber deformation variable in the figure is equal to the distance between the lowest point of the optical fiber center after deformation in the initial state and under pressure. The maximum deformations of the fiber in the semicircle-shape, square-shape, and V-shape structures are calculated by applying pressures of 0.5 MPa, 1 MPa, and 1.5 MPa in the simulation, whose results are shown in Figure 3d–f. It can be seen that the deformation in the square-shape structure is the maximum and the V-shape is the minimum under the same pressure, which means that the OMCSL in the square-shape structure has the maximum pressure sensitivity compared to other structures.

## 4. Experiment Results

### Analysis of Pressure Sensing Characteristics in Different Package Structures

**Figure 5:**
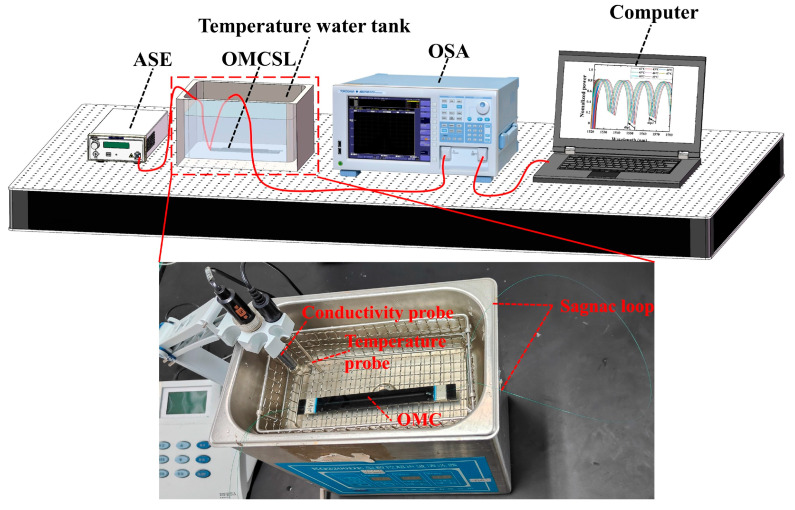
The schematic diagram of the temperature experiment system.

In temperature experiments, sensors with the same parameters should be used. The packaging structure of the slot is different. In this paper, temperature sensing tests are performed by the system in Figure 5, which consists of an ASE broadband light source (C + L band ASE Broadband Light Source, ASE-CL-100-T-B), an optical spectrum analyzer (OSA, 600–1700 nm, resolution 0.002 nm, AQ63700, YOKOGAWA, Tokyo, Japan), and a temperature water tank (KQ2200DE, Kunshan Ultrasonic Instruments, Kunshan, China) with seawater and a conductivity probe (DDSJ-308A, Leici, Shanghai, China).

In this paper, in order to avoid errors caused by uneven heating of seawater during the heating process, the setting of the temperature water tank is first set to 50 °C. Then, the heating of the temperature water tank causes the seawater temperature to exceed 50 °C at the beginning, and stops when the temperature probe reaches 52 °C, and finally, it waits for the seawater to cool down naturally. During this cooling process of the seawater, the seawater only exchanges heat with the air and water tank, and the cooling process is stable and uniform. The temperature probe indication is observed when the seawater is first cooled down to 50 °C, and then the spectrum is collected by the OSA in steps of 1 °C until the experiment is completed.

**Figure 6:**
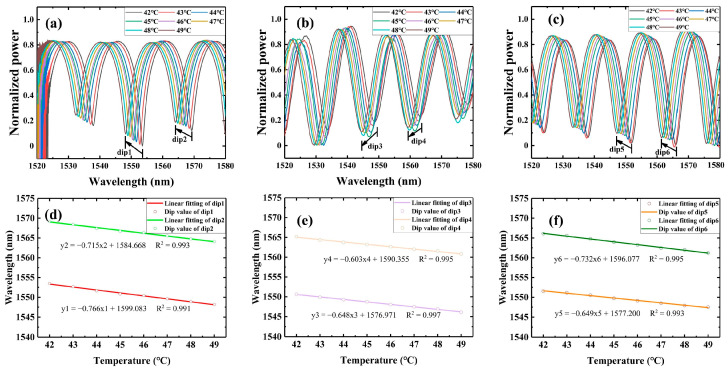
Reflection spectra of (**a**) V-shape, (**b**) square-shape, and (**c**) semicircle-shape sensors at different temperatures. The characteristic wavelength shifts at different temperature for (**d**) dip 1 and dip 2, (**e**) dip 3 and dip 4, and (**f**) dip 5 and dip 6.4.2. Pressure Sensing Experiment.

The decrease in seawater temperature causes a decrease in the PDMS refractive index, which in turn causes a decrease in the effective refractive index of the super mode inside the OMCSL under the effect of the evanescent field, eventually leading to a blueshift of the interference peak, as shown in Figure 6a–c. The temperature sensitivity can be obtained by calculating the characteristic wavelength shifts at different temperatures; the results are shown in Figure 6a–f. The temperature sensitivities of the OMCSL in V-shape, square-shape, and semicircle-shape package structures are −0.766 nm/°C, −0.648 nm/°C, and −0.649 nm/°C near 1550 nm, respectively, and −0.715 nm/°C, −0.603 nm/°C, and −0.732 nm/°C near 1565 nm, respectively. In the temperature range discussed in this paper, the difference in temperature sensitivity of the sensors depends mainly on the structural parameters of the fiber and the ratio of the PDMS solution. Different shapes of the PDMS do not affect the temperature sensitivity, so the OMCSLs in different package structures have essentially the same temperature sensitivity.

**Figure 7:**
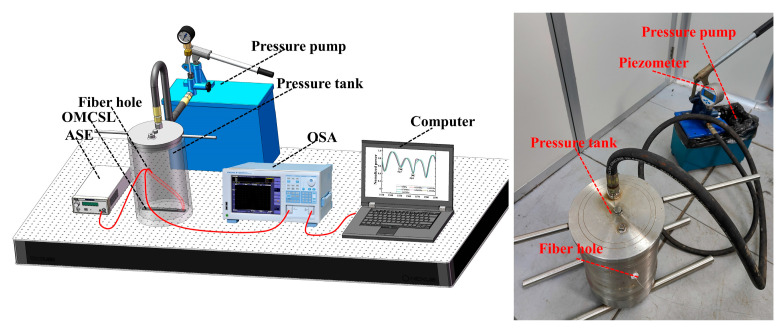
The schematic diagram of the pressure experiment system.

Pressure experiments are conducted to verify the simulation results. The experimental structure is shown in Figure 7: different OMCSLs are put into a pressure tank, and a piezometer (YB80A, YBPCM) and a pressure pump (SY-25, Hengqi, Shanghai, China) are used to measure pressure in the pressure tank in real time in order to test its real spectral response to water pressure.

The pressure is set in steps of 0.25 MPa and a pressure pump is used to pressurize seawater in the pressure tank. The pressure on the piezometer is observed when pressure is increased by about 0.25 MPa, and then the spectrum of the current pressure is collected by the OSA. Then, the above experimental steps are repeated until the pressure reaches 1.5 MPa. The pressure sensitivity of the dip or peak is calculated in different wavelengths after pressure tests of the different of package shape structures. With increasing pressure, the characteristic wavelength of the reflection spectrum shifts linearly in the long-wave direction due to the combined effect of the PDMS elastomeric effect and the deformation of the OMCSL waist region. In addition, the shift of the characteristic wavelength at different pressures can be calculated to obtain the pressure sensitivity.

**Figure 8:**
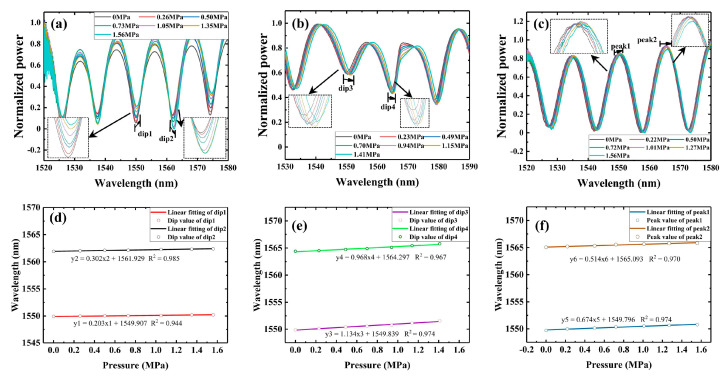
Reflection spectra of (**a**) V-shape, (**b**) square-shape, and (**c**) semicircle-shape sensors at different pressures. The characteristic wavelength shifts at different pressure for (**d**) dip 1 and dip 2, (**e**) dip 3 and dip 4, and (**f**) peak 1 and peak 2.

The calculated and fitted results are shown in Figure 8. The pressure sensitivities of the OMCSL in V-shape, square-shape, and Semicircle-shape package structures are 0.203 nm/MPa, 1.135 nm/MPa, and 0.674 nm/MPa near 1550 nm, respectively, and 0.302 nm/MPa, 0.968 nm/MPa, and 0.514 nm/MPa near 1565 nm, respectively. The pressure sensitivity of the OMCSL varies greatly depending on the package structure, mainly due to the different support effects of each structure on the OMCSL, and the resulting differences in force distribution. As a result, the square-shape structure has the largest average distance from the fiber to the bottom of the slot, giving it the highest pressure sensitivity, followed by the semicircle-shape, and the V-shape with the lowest sensitivity. However, in real-world environments, a PDMS layer that is too thin may fail to provide adequate protection for the fiber. Despite this, the experimental results are consistent with the simulation results, suggesting that the square-shape structure can be utilized to enhance pressure sensitivity in future sensor package designs.

## 5. Ill-Conditioned Matrix Discussion

Based on the above experimental results, the proposed sensor responds well to temperature and pressure changes. However, when the temperature and pressure change simultaneously, the proposed sensor is needed to handle the cross-sensitivity problem. Typically, the SMM is used to deal with this problem, and this method involves a standard sample with some known initial parameters to calibrate the system in the experiment, which is as follows:

(5) $$\left[\begin{array}{c} T-T_{0}\\ P-P_{0} \end{array}\right]={\left[\begin{array}{cc} S_{T_{1}} & S_{P_{1}}\\ S_{T_{2}} & S_{P_{2}} \end{array}\right]}^{-1}\left[\begin{array}{c} \lambda_{\mathrm{dip}1}-\lambda_{10}\\ \lambda_{dip2}-\lambda_{20}^{} \end{array}\right],$$

where, $\lambda_{10}$ and $\lambda_{20}$ are the initial wavelengths of dip1 and dip2 at the initial temperature and pressure of $\left(T_{0},P_{0}\right)$, respectively. The following equation can be obtained by transforming Equation (5):

(6) $$S_{T_{1}}T+S_{P_{1}}P=\lambda_{dip1}-\lambda_{10}+S_{T_{1}}T_{0}+S_{P_{1}}P_{0},$$

When $\lambda_{dip1}$ and $\lambda_{dip2}$ are input to the matrix as measured data, Equation (5) equals to a constant. Then, the sensitivity matrix can be considered as a linear system of equations with T and P as variables. This means that the intersection of two diagonal lines is the solution, which corresponds to the temperature and pressure parameters.

**Figure 9:**
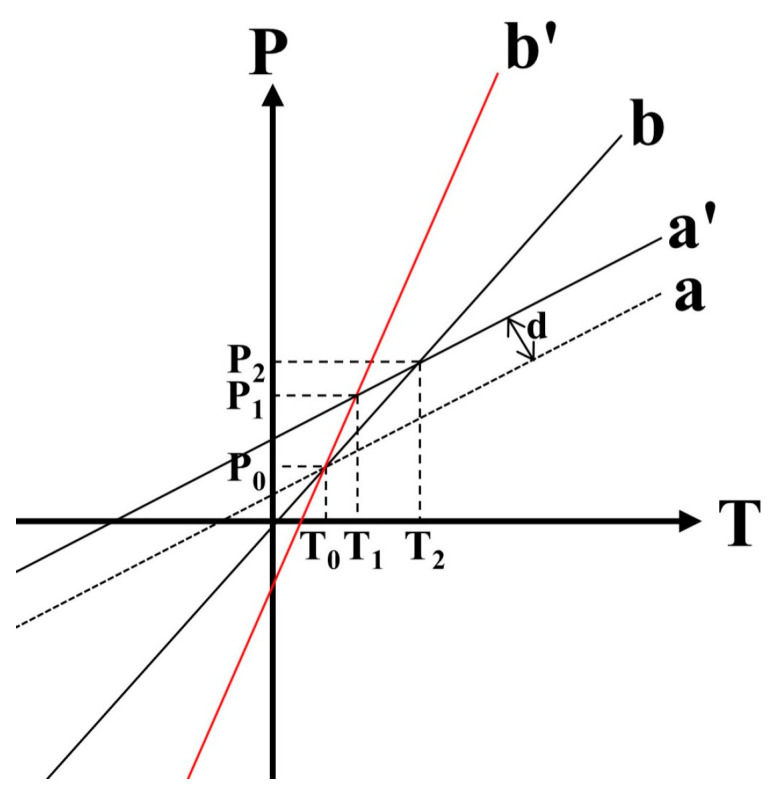
Comparison of the error between different angles.

However, in the SMM, the temperature and pressure sensing characteristics in a single sensor are basically equal, so that the difference of angle between the two diagonal lines is too small, which results in a small input error that has a large impact on the output result; a matrix with this problem is called an ill-conditioned matrix. As shown in Figure 9, for the sensitivity matrix of a sensor, the angle between line a and line b in the matrix is small at first. After changing the sensing characteristics of a sensor, line b will be rotated by a certain angle and turn into line $b^{\prime }$, and the angle becomes larger. In practice, the data are subject to errors caused by the resolution of the observation equipment, manufacturing errors, and the observation environment, and it is assumed that these errors make line a move a fixed distance d along the y-axis, and turn line a into line $a^{\prime }$. As can be seen in the figure after that, the intersection of line a and line b changes from $\left(T_{0},P_{0}\right)$ to $\left(T_{2},P_{2}\right)$ under the influence of the error, but the intersection is $\left(T_{1},P_{1}\right)$ in a larger angle system with line $b^{\prime }$. Obviously, for the same error, the difference between $\left(T_{2},P_{2}\right)$ and the true value $\left(T_{0},P_{0}\right)$ obtained from the demodulation of the two lines with a smaller angle is larger than that obtained from the one with a larger angle $\left(T_{1},P_{1}\right)$. In this case, the illness degree is introduced to describe the degree of the ill-conditioned matrix. The larger the corresponding angle of the matrix, the smaller the illness degree; the smaller the angle, the larger the illness degree. Among these, when the angle is too large that the two lines are perpendicular to each other, this means that the physical quantities corresponding to the two lines have no cross-sensitivity at all, and a change in one of the physical quantities has no effect on the other. As shown in Table 1, the temperature and pressure sensitivity of each wavelength will correspond to a line, and the angle between lines at different wavelengths corresponds to the illness degree of the matrix.

The values of the elements in the SMM are real data collected from experimental tests, so the problem of the ill-conditioned matrix cannot be solved by deformation of mathematical methods. Since different package structures do not affect the temperature sensitivity, but do affect the pressure sensitivity of the sensor, this paper proposes to change the package structure of the OMCSL to obtain a larger difference between temperature and pressure sensitivity, in order to alleviate the illness degree of the ill-conditioned matrix in the SMM.

Different package structures and structural parameters lead to the different sensitivities at different wavelengths, which are shown in the linear equations as different slopes of lines corresponding to different wavelengths. Different wavelengths correspond to different temperature and pressure sensitivities, so the dip or peak should be selected at a similar wavelength as much as possible; in this paper, the dip or peak at 1550 nm and 1565 nm are selected for measurement. The sensitivity is measured by the temperature and pressure experiments in Table 1.

As shown in the table, the semicircle-shape has the maximal angle difference, which implies that the matrix illness degree corresponding to the semicircle-shape is minimal when using the SMM; thus, using the semicircle-shape can effectively improve the ill-conditioned matrix problem. In addition, this paper illustrates the advantages of the semicircle-shape by comparing it with different package structures at the same error (1% F.S.). It is assumed that the wavelength input has the same error (wavelength range is 1520–1580 nm, so 1% F.S. is equivalent to 0.6 nm) to output the result, and this error may be caused by system error, environmental error, reading error, etc., which cannot be avoided. The effect on temperature and pressure under the influence of error can be calculated by combining Equations (5) and (6), as follow:

(7) $$\begin{array}{l} \Delta T=\frac{S_{P_{2}}\Delta \lambda_{dip1}-S_{P_{1}}\Delta \lambda_{dip_{2}}}{S_{T_{1}}S_{P_{2}}-S_{P_{1}}S_{T_{2}}}\\ \Delta P=\frac{S_{T_{2}}\Delta \lambda_{dip1}-S_{T_{1}}\Delta \lambda_{dip_{2}}}{S_{T_{1}}S_{P_{2}}-S_{P_{1}}S_{T_{2}}} \end{array},$$

where, $∆\lambda_{dip1}$ and $∆\lambda_{dip2}$ represent the difference between input wavelength and actual wavelength under the error, respectively, ∆T represents the difference between output temperature and actual temperature under the error, and ∆P represents the difference between output pressure and actual pressure under the error. The sensitivity of different sensors can be read from Table 1 as $S_{T_{1}},S_{T_{2}},S_{P_{1}},S_{P_{2}}$. The temperature and pressure errors were calculated to be 0.69 °C and 0.36 MPa for V-shape, 1.77 °C and 0.48 MPa for square-shape, amd 0.60 °C and 0.31 MPa for semicircle-shape. Although the semicircle-shape has the minimal temperature and pressure error with the same input error, the square-shape has the maximum pressure sensitivity; therefore, the package structure should be selected after considering the performance and practicality of the sensor in practice.

The above description shows that using a semicircle-shape package structure can significantly increase the angle between the lines in a sensitivity matrix and effectively reduces the error; thus, it can effectively optimize the ill-conditioned matrix problem. This result will provide a new method to solve the ill-conditioned matrix problem in SMM demodulation.

## 6. Comparison of Two Different Demodulation Methods

**Figure 10:**
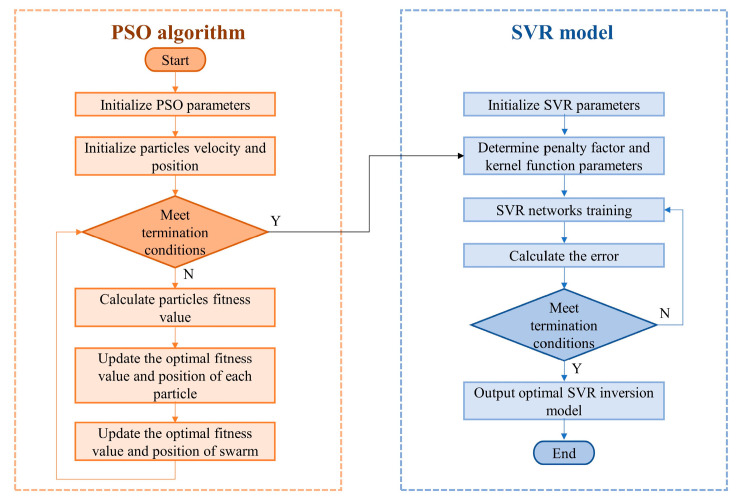
The algorithm flowchart of the PSO–SVR model.

When demodulating the temperature and pressure of a sensor, using the SMM will amplify the system error and make the error unacceptable. In this paper, the machine learning method (MLM) is used to avoid the error amplification caused by the SMM, and to further improve the measurement accuracy. In the case of a small sample size, the method of the support vector regression (SVR) model with particle swarm optimization (PSO) is calculated, the flowchart of which is shown in Figure 10, and compared with the data obtained by a temperature and depth compact logger (TD) to calculate the mean absolute error (MAE) and mean absolute percentage errors relative to the full scale (MAPE). Before the experiment, 257 sets of spectra under different temperature and depth conditions were collected, where 80% of the spectral data were randomly placed into the training set for model training, and the remaining 20% were placed into the test set for validation to avoid data chance.

**Figure 11:**
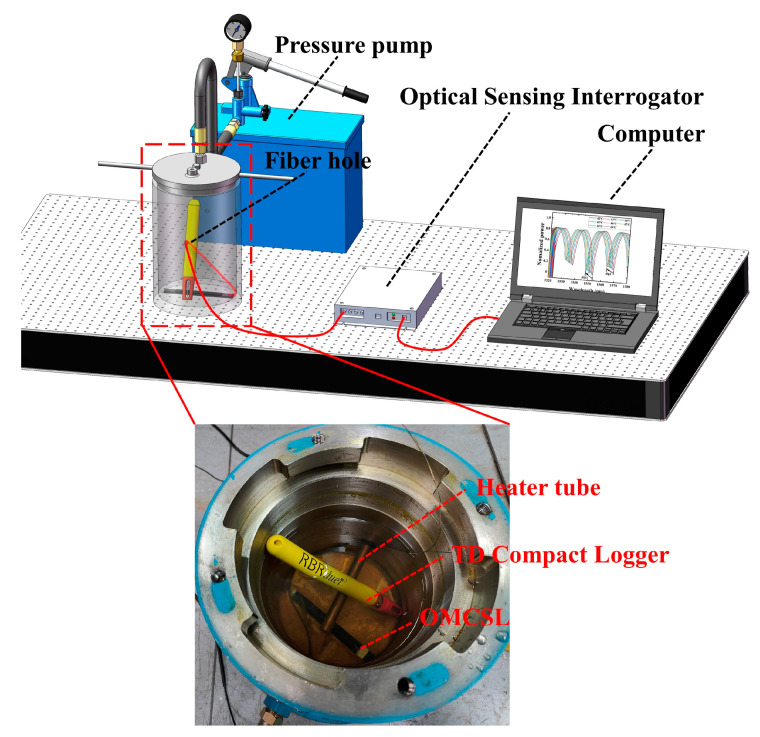
The schematic diagram of the variable temperature and pressure conditions experiment system.

In order to compare the accuracy of MLM predictions with the SMM, the OMCSL is placed in a pressure tank, and the temperature and pressure are controlled by using a pressure pump and a heater tube, the experimental setup of which is shown in Figure 11. A temperature and depth compact logger (TD, RBRdurt3, RBR, Ottawa, Ontario, Canada) is used to measure the temperature and pressure in the tank. Due to the loop structure of the sensor, light is emitted from the optical sensing interrogator (OSI, GAOSIGUANGDIAN, Wuhan, China) and returned to the OSI by the Sagnac loop. Then, the OSI transmits the obtained spectral information to the computer, where an algorithm can be run to output the temperature and pressure under the current experimental conditions in real time.

The comparison results are shown in Table 2, which shows that if the data collected by the TD compact logger is used as the standard value, the temperature measurement range is 15–50 °C, and the pressure range is 0–15 MPa. The measured temperature and pressure MAE are 4.98 °C and 1.92 MPa for the SMM, and 2.42 °C and 0.72 MPa for the MLM, respectively. The temperature and pressure MAPE are 14.23% and 16.13% for the SMM, and 5.49% and 4.80% for the MLM, respectively. Therefore, the MLM has higher prediction accuracy; different MLMs have different accuracy optimization effects, and the follow-up should compare several different MLMs to choose the best method. The SMM is simple and does not require a complicated modeling training process, and if there is no higher requirement for accuracy in an engineering practice, the SMM can be considered.

## 7. Conclusions

In this paper, three different package structures, V-shape, square-shape, and semicircle-shape, are designed and fabricated, an OMCSL is encapsulated by PDMS into these structures, and the sensing response characteristics of the OMCSL under different package structures are discussed. This paper also analyzes the temperature and pressure response characteristics of the OMCSL under different package structures by simulation and experiment. The experimental results show that the pressure sensitivities of the OMCSL are 0.203 nm/MPa, 1.135 nm/MPa, and 0.674 nm/MPa for the V-shape, square-shape, and semicircle-shape package structures, respectively, so the square-shape package structure has the maximum pressure sensitivity; the temperature sensitivities are −0.766 nm/°C, −0.648 nm/°C, and −0.649 nm/°C, respectively, so the temperature sensitivity essentially does not change with the change of package structure. In addition, with an input error of 1% F.S., the temperature and pressure errors are calculated to be 0.69 °C and 0.36 MPa for V-shape, 1.77 °C and 0.48 MPa for square-shape, and 0.60 °C and 0.31 MPa for semicircle-shape, respectively. Compared with the V-shape and square-shape structures, the semicircle-shape structure can significantly increase the angle between the lines in the sensitivity matrix and reduce the influence of input error, thereby effectively improving the ill-conditioned matrix problem. Finally, this paper also compares the demodulation error using the SMM and MLM; the MLM has higher demodulation accuracy, so different methods should be selected according to the actual engineering needs. In conclusion, this paper proposes using different packaging structures to effectively improve pressure sensitivity, introduces the concept of the ill-conditioned matrix, and provides a new approach to solve the ill-conditioned matrix problem in SMM demodulation. This essentially illustrates the optimization direction for solving the cross-sensitivity problem of transmitting multiple parameters, which is of practical engineering significance.

Based on the results of the above experiments, it has been demonstrated that different encapsulation structures can effectively address the ill-conditioned matrix problem in the SMM. Building on this research, further optimization can be pursued with the following ideas in mind:

(a) The sensor’s packaging structure and the structural parameters of the OMCSL have a significant impact on temperature and pressure sensitivity. Future experiments can explore the package thickness of the sensor, the diameter of the waist area, and the coupling length of the OMCSL. Through stepwise optimization of the structure, an optimal OMCSL sensing configuration can be developed.

(b) For the MLM, a large amount of real-world sensor data needs to be collected to improve demodulation accuracy. The number of samples should be increased, and the impact of different algorithms on prediction accuracy should be examined to determine the most suitable algorithm for specific practical scenarios.

## Figures and Tables

**Table 1:** Comparison of fiber optic sensitivity with different wavelengths.

	V−Shape		Square−Shape		Semicircle−Shape	
	1550 nm	1565 nm	1550 nm	1565 nm	1550 nm	1565 nm
Temperature sensitivity (nm/°C)	−0.766	−0.715	−0.648	−0.602	−0.649	−0.732
Pressure sensitivity (nm/MPa)	0.203	0.302	1.135	0.968	0.674	0.514
Tilt angle (°)	75.1	67.1	29.7	31.9	43.9	54.9
Angle difference (°)	8.0		2.2		11.0	

**Table 2:** Comparison of temperature and pressure with different methods.

No.	Temperature (°C)			Pressure (MPa)		
	TD	SMM	MLM	TD	SMM	MLM
1	29.11	25.79	31.56	3.70	1.33	3.01
2	31.62	37.05	30.32	7.84	10.34	7.52
3	32.52	27.58	35.31	5.10	8.57	5.96
4	28.79	23.97	30.74	2.13	0.69	2.49
5	30.99	24.56	32.45	8.33	6.39	9.26
6	35.48	40.3	32.37	4.84	2.54	3.87
7	38.77	32.41	40.21	0.48	2.37	1.50
8	40.70	43.36	40.15	7.07	9.93	8.12
9	29.23	35.89	26.41	5.43	2.82	4.89
10	35.32	39.67	36.64	6.82	4.02	7.27
MAE	0	4.98	1.92	0	2.42	0.72
MAPE (%)	0	14.23%	5.49%	0	16.13%	4.80%

## Data Availability

The data presented in this study are available on request from the corresponding author.
